# Effect of Sex and Adaptation on Migraine Frequency and Perceived Stress: A Cross-Sectional Case-Control Study

**DOI:** 10.3389/fneur.2019.00598

**Published:** 2019-06-05

**Authors:** Yu-Chin An, Chih-Sung Liang, Jiunn-Tay Lee, Meei-Shyuan Lee, Sy-Jou Chen, Chia-Lin Tsai, Guan-Yu Lin, Yu-Kai Lin, Fu-Chi Yang

**Affiliations:** ^1^Department of Emergency Medicine, Tri-Service General Hospital, National Defense Medical Center, Taipei, Taiwan; ^2^Department of Psychiatry, Beitou Branch, Tri-Service General Hospital, National Defense Medical Center, Taipei, Taiwan; ^3^Department of Neurology, Tri-Service General Hospital, National Defense Medical Center, Taipei, Taiwan; ^4^School of Public Health, National Defense Medical Center, Taipei, Taiwan

**Keywords:** perceived stress, headache, migraine, frequency, adaptation, sex difference

## Abstract

**Background:** Perceived stress has been related to migraine. The relationship between sex, migraine frequency, and severity of perceived stress remains unclear. We investigated perceived stress among migraineurs.

**Methods:** This cross-sectional case-control study involved 577 clinical outpatients at a tertiary hospital in Taiwan. Demographic and clinical data, including migraine characteristics, were collected. Migraineurs were stratified by episode frequency, aura and sex, and analyses were controlled for confounding variables. Multivariable linear regressions were used to inspect whether migraine frequency (1–4, 5–8, 9–14, or ≥15 headache days per month) was associated with perceived stress as assessed by the Perceived Stress Scale (PSS).

**Results:** Perceived stress was significantly higher in high frequency migraineurs (mean ± standard deviation (SD), 23.3 ± 8.7) than in low frequency migraineurs (mean ± SD, 21.9 ± 9.2; *P* < 0.05). After stratifying the analysis by sex, this result was observed in male subjects, but was insignificant in female subjects. In addition, the relationship between migraine frequency and perceived stress was not prominent in aura-present or -absent subgroups.

**Conclusions:** Higher perceived stress was associated with higher migraine frequency, but not in chronic migraine and female subgroups. Adaptation to migraine and various psychiatric comorbidities may contribute to these associations.

## Background

Migraine is an important public health issue due to its high prevalence, affecting more than one billion people worldwide (1–4). As ranked by the World Health Organization, headache disorders are among the top 10 disabling conditions for both sexes and among the top five most disabling disorders for women (5). Since childhood, headache affects about 60% of children and adolescents worldwide, thus affecting school, physical activities, peer and family relationships. (6) Several triggers may increase migraine frequency, such as (1) variations in hormones; (2) changes in weather, meals, caffeine, medication, obesity, sleep quality, and (3) stressful events (7). Mounting evidence indicates that several comorbidities, such as anxiety, depression, sleep disturbances, vascular accidents, epilepsy, restless legs syndrome, and stress, are associated with migraine (4, 8, 9). In addition, a high prevalence of psychiatric comorbidities in patients with episodic and chronic headache, especially for anxiety and affective disorders has been reported (10). On the other hand, severe migraine attacks also may worsen these conditions (11).

Stress is an adaptive response to the perceived demands created by physical or psychological stimuli (12). Cohen et al. (13) created the perceived stress scale (PSS) to measure the levels to which situations in one's life are considered stressful. In subsequent decades, several studies have indicated that perceived stress is associated with multi-morbidity, mental illness, lower quality of life, and even increased mortality (14–16). Additionally, Guidetti et al. indicated that headache develops when humans face strong sensory stimuli, and physical or emotional stress might be the result of an evolved defense mechanism as a mediator of the environmental stressors and psychological factors (17). Previous studies have demonstrated that migraineurs have higher levels of perceived stress than controls (18). Perceived stress is considered to precipitate, exacerbate and perpetuate migraine (18, 19). Higher perceived stress levels are considered to occur more frequently in female than male migraineurs (12). Collectively, stress is commonly associated with migraine.

Although the relationship between migraine and stress is reliable, to our knowledge, currently no studies have definitively shown a positive trend between migraine frequency and perceived stress intensity. Furthermore, a previous study found no difference in mean PSS scores between migraineurs and controls after adjusting for depression/anxiety (19), suggesting that perceived stress in migraineurs could be affected by other factors, including depression/anxiety, that remain to be investigated. In addition, the difference in perceived stress between migraineurs with or without aura remains unclear.

Therefore, we tested the hypothesis that perceived stress intensity may predict migraine frequency, regardless of the presence of auras. In particular, we investigated the relationship between perceived stress intensity and migraine frequency while controlling for potentially confounding factors, such as sleep quality, employment status, education, smoking status, alcohol intake, coffee consumption, and depression/anxiety.

## Methods

### Patients

Our cross-sectional study enrolled 557 subjects visiting an outpatient headache clinic in the Department of Neurology at Tri-Service General Hospital between January 2016 and August 2018. All participants signed a written informed consent form after a full written and verbal explanation of the study. The study protocol was approved by the Institutional Review Board of the TSGH (No. 2-106-05-163). Patients with migraine, of both sexes, with and without auras, were enrolled after giving informed consent according to International Headache Society criteria (20). Among the study subjects, 100 experienced chronic, 97 high, 93 medium, and 174 low frequency migraine (>15, 9–14, 5–8, and 1–4 headache days/month, respectively). In addition, 93 volunteers without migraine, who had no family history of migraine and no previous diagnosis of other primary or secondary headache disorders, except for episodic tension-type headaches (<6 days/year), were selected.

Each participant who completed the screening questionnaire was interviewed by a board-certified neurologist and headache specialist (F-CY) to confirm the migraine diagnosis and exclude emotion-related headaches, as determined according to the International Classification of Headache Disorders, 3rd edition (beta version) (21, 22).

### Psychometrics Analysis

Patients were interviewed with a structured questionnaire packet containing the PSS (13), Beck Depression Inventory (BDI) (23), Hospital Anxiety and Depression Subscales (HADS) (24), restless legs syndrome (RLS) screening questionnaire from the RLS Foundation (25), Pittsburgh Sleep Quality Index (PSQI) (26), and Migraine Disability Assessment questionnaire (MIDAS) (27). The PSS, a 14-item scale with seven positively stated items that are scored in reverse direction, was used to measure perceived stress. Each item is rated on a five-point scale (0, never; 1, almost never; 2, sometimes; 3, fairly often; 4, very often). Patients who scored ≥18 on the BDI (maximum score = 63) were classified as depressed. The HADS is a 14-item scale, with seven items related to anxiety and seven related to depression, each rated on a four-point scale (0, not at all; 1, sometimes; 2, often and 3, all the time), giving a maximum subscale score of 21. The PSQI estimates sleep quality over the prior month, with a final score of 6 indicating poor sleep. Participants who answered “yes” to at least 6/11 RLS symptom screening questionnaire items were considered to have a high probability of RLS. The MIDAS, a 5-item questionnaire, evaluated disability over the previous 3 months.

### Statistical Analysis

Continuous and categorical data were expressed as mean ± standard deviation (SD) and frequency and proportion, respectively. Patients with migraines were classified into four ordinal migraine frequency groups. Linear trends in variable distributions were assessed across the control and ordinal migraine frequency groups using the Cochran-Armitage χ^2^ test for categorical variables or a linear contrast of general linear model for continuous variables. The PSS total score was pairwise compared among the four migraine frequency groups using a multivariable linear regression analysis adjusted for all the variables listed in Table 1. The multivariable linear regression analysis was stratified further by aura and sex. Finally, we conducted a multivariable linear regression analysis to explore factors associated with PSS total score in the patients with migraine. Differences with a 2-sided *P* < 0.05 were considered statistically significant. No adjustment for multiple testing (multiplicity) was made in this study. Statistical analyses were conducted using SPSS 22 (IBM SPSS, Inc., Armonk, NY, USA).

**Table 1:** Characteristics of the study population (*N* = 557).

**Variable**	**Control**	**The migraine group**				***P* trend**
		**Low (1–4 days)**	**Medium (5–8 days)**	**High (9–14 days)**	**Chronic (≥15 days)**	
Patient number	93	174	93	97	100	–
Migraine with aura	–	51 (29.3)	32 (34.4)	26 (26.8)	43 (43.0)	0.081
Female sex	70 (75.3)	123 (70.7)	62 (66.7)	73 (75.3)	79 (79.0)	0.326
Age (years)	38.2 ± 10.9	38.4 ± 11.5	36.0 ± 11.4	36.6 ± 11.9	37.7 ± 11.5	0.452
BMI (kg/m^2^)	23.3 ± 3.7	23.0 ± 3.5	23.1 ± 4.2	22.6 ± 3.3	22.9 ± 4.1	0.310
Education level (years)	14.1 ± 2.5	14.8 ± 2.2	14.5 ± 2.9	14.3 ± 2.9	14.1 ± 2.7	0.541
Marital status						0.135
Single	38 (40.9)	70 (40.2)	39 (41.9)	53 (54.6)	44 (44.0)	
Married	48 (51.6)	91 (52.3)	51 (54.8)	39 (40.2)	50 (50.0)	
Divorced or widowed	7 (7.5)	13 (7.5)	3 (3.2)	5 (5.2)	6 (6.0)	
Employment status						0.259
Unemployed	4 (4.3)	8 (4.6)	3 (3.2)	5 (5.2)	8 (8.0)	
Has a job	72 (77.4)	141 (81.0)	81 (87.1)	83 (85.6)	75 (75.0)	
Retired or household	17 (18.3)	25 (14.4)	9 (9.7)	9 (9.3)	17 (17.0)	
Smoking	17 (18.3)	30 (17.2)	25 (26.9)	21 (21.6)	23 (23.0)	0.218
Alcohol intake	26 (28.0)	69 (39.7)	43 (46.2)	34 (35.1)	38 (38.0)	0.414
Coffee consumption						0.576
Never	25 (26.9)	31 (17.8)	16 (17.2)	19 (19.6)	27 (27.0)	
< once a month	19 (20.4)	40 (23.0)	24 (25.8)	29 (29.9)	19 (19.0)	
≥1 day a week	49 (52.7)	103 (59.2)	53 (57.0)	49 (50.5)	54 (54.0)	
MIDAS	–	15.9 ± 12.2	22.2 ± 16.0	23.7 ± 16.8	31.4 ± 20.8	< 0.001
BDI total score	7.0 ± 5.3	9.7 ± 7.6	9.4 ± 7.2	10.6 ± 9.3	13.3 ± 9.3	< 0.001
HADS–anxiety	6.1 ± 3.8	7.1 ± 4.4	7.7 ± 4.2	7.9 ± 4.5	9.2 ± 4.4	< 0.001
HADS–depression	4.1 ± 3.0	4.9 ± 3.9	5.0 ± 3.4	6.2 ± 4.3	6.9 ± 4.4	< 0.001
PSQI total score	7.5 ± 3.7	8.1 ± 3.7	8.8 ± 3.6	8.6 ± 4.0	11.1 ± 3.8	< 0.001

*MIDAS, migraine disability assessment; BDI, Beck depression inventor; HADS, hospital anxiety and depression scale; PSQI, Pittsburgh sleep quality index. Continuous data are presented as mean ± standard deviation*.

## Results

### Characteristics of the Study Subjects

Table 1 lists the characteristics of subjects in the control (93, 16.7%) and migraine frequency (464, 83.3%) groups. There was no significant difference in terms of aura, sex, age, body mass index (BMI), education level, marital status, employment status, smoking, alcohol drinking, or coffee consumption between the groups. The scores for the migraine disability, anxiety, depression, and sleep quality assessments worsened as migraine frequency increased (*P* < 0.001).

### PSS Total Score

Table 2 lists the descriptive statistics for items on the PSS scale in the control and migraine frequency groups. Except for items 1 and 12, the scores of the 12 items on the PSS scale increased as migraine frequency increased (*P* < 0.05). With regard to total score, perceived stress was correlated positively with a higher frequency of migraine (*P* < 0.001). Noticeably, there was no significant difference in total PSS score between the two groups (*P* > 0.99, Bonferroni multiple comparison, data not shown).

**Table 2:** PSS scale items in the control and migraine frequency groups.

**Item**	**Control**	**The migraine group**				***P* trend**
		**Low (1–4 days)**	**Medium (5–8 days)**	**High (9–14 days)**	**Chronic (≥15 days)**	
1. Upset because of something that happened unexpectedly	1.47 ± 0.94	1.63 ± 1.11	1.67 ± 1.06	1.53 ± 1.10	1.75 ± 1.01	0.176
2. Felt that you were unable to control important things in your life	0.91 ± 0.85	1.25 ± 1.09	1.26 ± 1.12	1.14 ± 1.04	1.45 ± 0.99	0.003
3. Felt nervous and “stressed”	1.33 ± 1.00	1.53 ± 1.09	1.63 ± 1.09	1.56 ± 1.11	1.81 ± 1.09	0.004
4. Dealt successfully with irritating life hassles^*^	1.62 ± 1.18	1.63 ± 1.13	1.85 ± 1.13	1.97 ± 1.07	1.95 ± 1.03	0.005
5. Felt that you were effectively coping with important changes that were occurring in your life^*^	1.57 ± 1.16	1.51 ± 1.09	1.75 ± 1.10	1.92 ± 1.06	1.88 ± 1.02	0.003
6. Felt confident about your ability to handle your personal problems^*^	1.39 ± 1.19	1.48 ± 1.14	1.54 ± 1.14	1.84 ± 1.10	1.78 ± 1.16	0.002
7. Felt that things were going your way^*^	1.80 ± 1.05	1.83 ± 1.04	2.05 ± 1.02	2.23 ± 1.05	2.21 ± 0.99	< 0.001
8. Found that you could not cope with all the things that you had to do	1.02 ± 0.81	1.26 ± 0.97	1.17 ± 0.96	1.16 ± 1.03	1.43 ± 0.98	0.017
9. Able to control irritations in your life^*^	1.67 ± 1.08	1.69 ± 1.09	1.81 ± 1.10	2.02 ± 1.04	2.15 ± 1.12	< 0.001
10. Felt that you were on top of things^*^	1.74 ± 1.26	1.78 ± 1.22	1.85 ± 1.17	2.11 ± 1.22	2.25 ± 1.12	< 0.001
11. Angered because of things that happened that were outside of your control	1.07 ± 0.86	1.59 ± 1.04	1.45 ± 0.95	1.53 ± 1.08	1.53 ± 1.01	0.006
12. Found yourself thinking about things that you have to accomplish	1.65 ± 1.06	1.39 ± 1.00	1.10 ± 0.94	1.38 ± 1.06	1.36 ± 0.92	0.067
13. Able to control the way you spend your time^*^	1.25 ± 1.02	1.45 ± 0.99	1.45 ± 1.03	1.63 ± 1.05	1.74 ± 1.01	< 0.001
14. Felt difficulties were piling up so high that you could not overcome them	0.99 ± 0.85	1.28 ± 1.02	1.42 ± 1.07	1.41 ± 1.10	1.58 ± 0.97	< 0.001
Total score	19.4 ± 8.2	21.2 ± 9.5	21.9 ± 9.2	23.3 ± 8.7	24.8 ± 9.4	< 0.001

*PSS, Perceived stress scale. 0, never; 1, almost never; 2, sometimes; 3, fairly often; 4, very often. Data are presented as mean ± standard deviation*.

* *Items 4, 5, 6, 7, 9, 10, and 13 are scored in reverse direction*.

**Figure 1:**
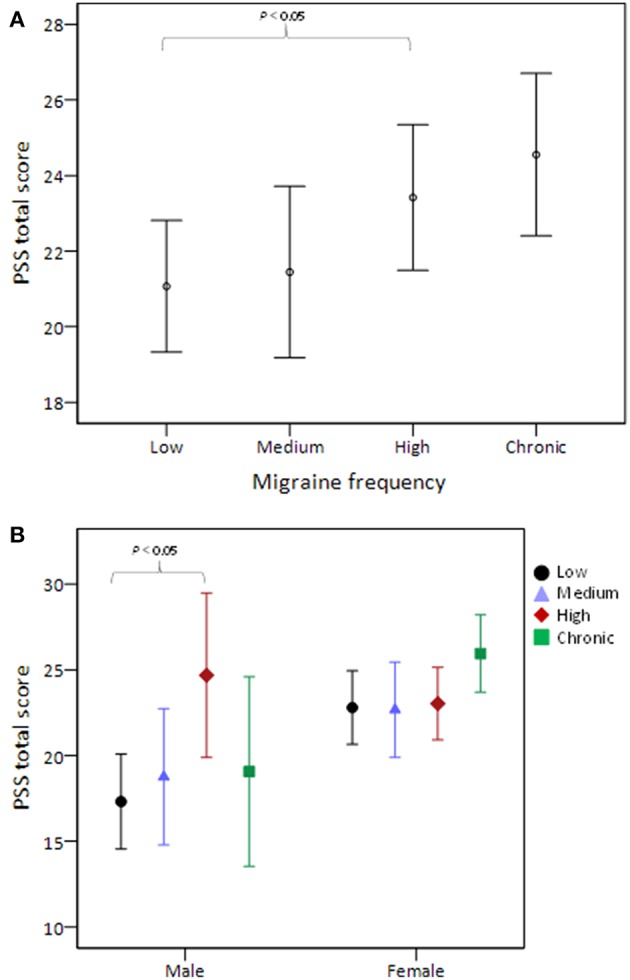
PSS total score stratified by migraine frequency **(A)** and the comparison was further stratified by sex **(B)**. The middle horizontal line represents the mean and the error bars represent 95% confidence interval around the means. PSS, Perceived Stress Scale.

Table 3 shows the pairwise comparisons of PSS total scores among the four migraine frequency groups with adjustment for all variables listed in Table 1. Perceived stress was significantly higher in high than in low frequency migraineurs (*P* = 0.035, Figure 1A). After stratifying this analysis by sex, this phenomenon was observed only in male (*P* = 0.04) and not in female (Figure 1B) subjects. In addition, the presence or absence of aura did not modify the association between migraine frequency and perceived stress.

**Table 3:** PSS total score in the migraine frequency groups stratified by sex and aura.

**Population/aura**	**The migraine group**			
	**Low (1–4 days)**	**Medium (5–8 days)**	**High (9–14 days)**	**Chronic (≥15 days)**
**The whole cohort**				
With or without aura	21.2 ± 9.5	21.9 ± 9.2	23.3 ± 8.7**^§^**	24.8 ± 9.4
With aura	21.5 ± 9.2	24.8 ± 10.6	23.5 ± 7.1	27.4 ± 7.1
Without aura	21.2 ± 9.6	20.4 ± 8.0	23.3 ± 9.3	22.8 ± 10.5
**Male**				
With or without aura	18.4 ± 9.2	20.0 ± 8.9	24.8 ± 9.1**^§^**	21.0 ± 9.9
With aura	19.6 ± 8.5	23.6 ± 8.6	24.0 ± 4.2	30.0 ± 4.7
Without aura	17.8 ± 9.6	18.5 ± 8.8	25.0 ± 10.4	18.9 ± 9.7
**Female**				
With or without aura	22.4 ± 9.4	22.9 ± 9.2	22.8 ± 8.6	25.8 ± 9.1
With aura	22.4 ± 9.6	25.3 ± 11.4	23.4 ± 7.9	27.2 ± 7.3
Without aura	22.4 ± 9.4	21.4 ± 7.4	22.7 ± 8.9	24.4 ± 10.5

*PSS, Perceived stress scale*.

*Adjusted for aura, sex, age, BMI, education level, marital status, employment status, smoking, alcohol drinking, coffee consumption, MIDAS, BDI total score, HADS–anxiety, HADS–depression and PSQI total score*.

§ *indicates significant difference (P < 0.05) vs. the low migraine frequency group*.

### Factors Associated With PSS Total Score

Table 4 lists the results of the multivariable linear regression analysis investigating factors associated with PSS total score. Multivariable analysis identified the following variables as independent factors: high (versus low) migraine frequency, younger age, higher perceived depression levels and higher perceived anxiety levels.

**Table 4:** Multivariable linear regression analysis of factors associated with PSS scores in the patients with migraine.

**Variable**	***B* (95% of CI)**	***P***
**Group of migraine frequency**		
Low (1–4 days)	Reference	–
Medium (5–8 days)	−0.06 (−1.87, 1.74)	0.944
High (9–14 days)	2.01 (0.14, 3.89)	0.035
Chronic (≥15 days)	0.45 (−1.55, 2.45)	0.657
Migraine with aura	0.20 (−1.24, 1.64)	0.782
Female sex	0.68 (−1.02, 2.38)	0.432
Age (years)	−0.18 (−0.26, −0.09)	<0.001
Education level (years)	−0.26 (−0.55, 0.03)	0.081
BMI (kg/m^2^)	−0.08 (−0.27, 0.11)	0.425
**Marital status**		
Single	Reference	–
Married	0.36 (−1.46, 2.19)	0.694
Divorced or widowed	−1.90 (−5.04, 1.25)	0.236
**Employment status**		
Unemployed	Reference	–
Has a job	−1.18 (−4.40, 2.04)	0.471
Retired or household	−0.93 (−4.63, 2.78)	0.623
Smoking	−0.44 (−2.21, 1.34)	0.628
Alcohol drinking	0.68 (−0.76, 2.12)	0.352
**Coffee consumption**		
Never	Reference	–
< once a month	−0.20 (−2.23, 1.82)	0.844
≥1 day a week	−0.45 (−2.20, 1.31)	0.617
MIDAS	−0.01 (−0.05, 0.04)	0.818
BDI	0.48 (0.36, 0.61)	< 0.001
HADS–anxiety	0.61 (0.41, 0.82)	< 0.001
PSQI total score	0.15 (−0.06, 0.36)	0.157

*B, regression coefficient; CI, confidence interval; MIDAS, migraine disability assessment; BDI, Beck depression inventor; HADS, hospital anxiety and depression scale; PSQI, Pittsburgh sleep quality index; PSS, perceived stress scale. HADS–depression was omitted in the model because of its multicollinearity with BDI total score*.

## Discussion

In our study, after adjusting for aura, sex, age, BMI, education level, marital status, employment status, smoking, alcohol intake, coffee consumption, MIDAS, BDI total score, HADS–anxiety, HADS–depression, and PSQI total score, the perceived stress level was significantly higher in the high than in the low migraine frequency group. We also found a relationship between perceived stress and migraine frequency in the male but not in the female group. Furthermore, migraines with or without auras did not modify the association between migraine frequency and perceived stress. It is noteworthy that there were no significant differences in total PSS score between the high frequency and chronic migraine groups.

Earlier studies have reported that migraine can be triggered by stress (9, 28, 29). A higher level of perceived stress was found previously in migraineurs compared to non-migraineurs (12, 30). Hence, we initially expected to find a trend between migraine frequency and perceived stress levels. Surprisingly, our study revealed that the perceived stress level was significantly higher in the high frequency migraineurs than in the chronic migraine group. It is known that chronic migraineurs can have more mood events, sleep problems and a poorer quality of life than patients with migraine episodes or healthy patients (31, 32). In a prospective cohort study, stress was comorbid with migraine and major depression (33). Various psychosomatic and psychiatric disorders influenced perceived stress in migraineurs (34). The influence of stress in chronic migraineurs partially explained why we identified higher perceived stress levels in those who experienced migraine at a higher frequency, but this was not statistically significant in the chronic migraine group when we adjusted for all potential co-variables, including psychiatric comorbidities.

On the other hand, adaptive and maladaptive changes in the stress response also have been suggested as a mechanism underlying chronic migraine (35, 36). The hippocampus has a role in stress adaptation, and structural and functional changes in the hippocampus have been related to stressful events (36). In addition, migraine has been suggested to alter the hippocampus volume (37). A smaller hippocampal volume may result in persistent pain states (38). In a cross-sectional study of episodic migraine, a significantly larger bilateral hippocampal volume was found in the low frequency group than in the high frequency and healthy control groups. This suggests an initial adaptive plasticity related to migraine that becomes dysfunctional with increased frequency (39). The results between our high and low frequency migraine groups are consistent with this suggestion. However, another study demonstrated different results, indicating no differences in hippocampal volume between groups with a headache frequency of 1–2 or 8–14 days/month, and the volumes peaked at 5–7 days/month (40). Furthermore, our results opposed those of Moon et al., who reported higher PSS scores in chronic migraineurs (19). This discrepancy implies that the role of adaptation to persistent headache and stress with regard to different migraine frequencies remain controversial. Persistent pain may produce stress-like changes in hippocampal neurogenesis (41). We suggest that chronic migraineurs may adapt to the experience of migraine and may also be affected by other psychiatric comorbidities. These may be the reasons we did not observe a significantly higher perceived stress level in this group in our study. Thus, the ability to adapt to persistent headache may influence perceived stress in migraineurs. This may explain why we did not find a clear association between perceived stress and migraine frequency in this or prior studies.

While high frequency migraineurs had a significantly higher level of perceived stress in our study, we investigated whether sex differences existed. The influence of sex on stress and migraine is worthy of discussion. The biological response to stress is associated with activity of the hypothalamic-pituitary-adrenal (HPA) axis, which is regulated by the release of hypothalamic corticotropin releasing factor (CRF) (42). Dysregulation of CRF, which is regarded as a stress neuropeptide, is thought to contribute to the pathophysiology of stress-related disorders (43). Several prior studies have demonstrated sex differences in the response to stress (44–47). Increased acute HPA and autonomic responses have been found in men compared to women (34). Furthermore, women have been previously reported to have higher stress sensitivity and a higher risk for depression and anxiety disorders compared to men (48, 49). Table 3 shows that women had generally higher PSS scores in each migraine frequency group compared to men except for the high frequency group. As psychiatric comorbidities may influence the perceived stress level, our results implied that women had a poor response to stress and more psychosomatic and psychiatric problems, including depression and anxiety disorders, which may have contributed to the insignificant association between migraine and perceived stress.

Several strengths to our study included a large number of subjects, controlled study design, demographically similar groups, sex-differentiated subgroups, use of validated questionnaires, consideration of sleep quality, and anxiety/depression disorders, analysis of migraine subgroups (with or without auras) and robust statistical analysis. Nevertheless, there several limitations also are worth mentioning. First, we used a cross-sectional design, which limited our ability to confirm causal inferences. Future studies with the longitudinal design are necessary to explore the possible causal relationship. Second, our study population comprised patients from a single tertiary hospital, limiting the broad generalizability of our findings. In the future studies, we would involve multiple centers to enroll more patients, and a population-based study may also be warranted. Third, perceived stress was evaluated with a self-rated PSS rather than with an objective assessment. Furthermore, we used the version of the PSS with 14 items, which is one of the most frequently used tools that has great validity (50). However, several recent studies have discussed the use of modified versions of the PSS and demonstrated higher consistency and reliability in a 10-item version (51–53). Among the 14 items used in our study, scores on items 1 and 12 were not consistently greater as the migraine frequency increased. In future studies, we will evaluate different tools for greater reliability and consistency. Lastly, most high frequency and chronic migraineurs had been treated with migraine-preventing agents, such as calcium channel blockers, β-blockers, antiepileptic drugs, or even antidepressants, all of which may have effects on psychiatric comorbidities. For the benefit of the participants, we permitted use of the preventive interventions to improve their quality of life. In future studies, we will take preventive interventions into consideration and adjust for these as confounding variables.

## Conclusions

In conclusion, our findings revealed that the perceived stress level was significantly higher in patients with high than in those with low migraine frequency. This also was found in men, but it was not significant in those with chronic migraine or in women. Adaptation to migraine and psychiatric comorbidities may contribute to the association between migraine frequency and perceived stress level.

## Data Availability

The raw data supporting the conclusions of this manuscript will be made available by the authors, without undue reservation, to any qualified researcher.

## Author Contributions

Y-CA and F-CY participated in data collection, analyzed the data, and drafted the manuscript. C-SL, J-TL, M-SL, S-JC, C-LT, G-YL, and Y-KL participated in the study design, collected the data and helped to draft the manuscript. F-CY supervised the study, conceptualized, and designed the study and helped to draft the manuscript. All authors read and approved the final manuscript.

### Conflict of Interest Statement

The authors declare that the research was conducted in the absence of any commercial or financial relationships that could be construed as a potential conflict of interest.

## Abbreviations

PSS: Perceived Stress Scale
BDI: Beck depression inventor
HADS: hospital anxiety and depression scale
RLS: restless legs syndrome
PSQI: Pittsburgh sleep quality index
MIDAS: Migraine Disability Assessment questionnaire
HPA: hypothalamic-pituitary-adrenal
CRF: corticotropin releasing factor.
